# CMAP-Fusion: A cross-modal feature selection and model pruning framework for laboratory and imaging data

**DOI:** 10.1371/journal.pone.0346875

**Published:** 2026-04-24

**Authors:** Chong Liu, Lei Yang, Jinmeng Lei

**Affiliations:** 1 Senior Engineer, Liuzhou Women and Children’s HealthCare Hospital, Liuzhou, Guangxi, China; 2 Senior Engineer, Guangzhou Women and Children’s Medical Center Liuzhou Hospital, Liuzhou, Guangxi, China; 3 Associate Senior Technologist, Guangzhou Women and Children’s Medical Center Liuzhou Hospital, Liuzhou, Guangxi, China; Samsun University: Samsun Universitesi, TÜRKIYE

## Abstract

Cross-modal fusion of medical imaging and laboratory data is a key pathway for accurate diagnosis of diseases, yet it is constrained by issues such as the modal heterogeneity gap, accumulation of feature redundancy, and efficiency imbalance. Existing methods struggle to balance precision and clinical adaptability, and some rely on simulated data leading to limited generalization ability. To address these challenges, we propose the Cross-Modal Alignment-Pruning Fusion model (CMAP-Fusion), which achieves optimization through modular collaboration of “encoding alignment → redundant pruning → fusion prediction”: ViT-B/16 is used to complete imaging feature extraction and dimension alignment, the SmartTrim dynamic pruning module screens key features and reduces redundancy, and the Cross-Modal Transformer (CMT) mines deep associations between dual modalities. Experiments on the COVID-19 Radiography Dataset, ISIC Skin Cancer Dataset, and ChestX-ray14 Dataset demonstrate that the model achieves accuracies of 95.3%, 89.7%, and 93.6% respectively, representing an improvement of 3.1% to 4.1% compared with optimal baselines. Meanwhile, the number of parameters is reduced by 44.2%, computational complexity is decreased by more than 43%, and cross-modal similarity and feature sparsity are significantly superior to baselines. This model realizes the synergistic optimization of “precision-efficiency-generalization,” providing an efficient solution for medical cross-modal fusion. In the future, we will expand to multi-source modalities and multi-disease scenarios, strengthen clinical multi-center validation, further improve the model’s interpretability and clinical acceptance, and facilitate the lightweight deployment of medical AI.

## Introduction

Multimodal data fusion has become the core engine for promoting innovation in the field of medical artificial intelligence. It relies on the complementary information fusion mechanism of multi-source heterogeneous data to provide more comprehensive decision-making basis for key clinical tasks such as accurate disease diagnosis and prognostic risk assessment [1]. Clinical laboratory data (e.g., time-series quantitative indicators including blood routine parameters and inflammatory factor levels) possesses both objective quantifiability and dynamic monitoring value, enabling it to reflect dynamic changes in the body’s physiological and pathological states; in contrast, medical imaging data (e.g., chest CT scans and dermoscopic images) can intuitively depict the spatial morphology, anatomical location, and pathological feature distribution of lesions [2]. The deep fusion of the two can break through the information bottleneck of single-modal data and show irreplaceable application potential in clinical scenarios such as early disease screening and lesion qualitative classification [3,4]. However, laboratory data and imaging data have significant modal heterogeneity in data structure, feature distribution and semantic expression. This inherent difference makes it difficult to establish accurate semantic association in the process of cross-modal fusion.

Existing multimodal fusion methods still confront three key challenges in processing laboratory and image data. First, there exists a significant semantic gap between modalities. Laboratory time-series data and image spatial data exhibit distinct feature distributions, making it challenging for traditional methods to establish accurate cross-modal associations and resulting in insufficient discriminative power of fused features [5]. Second, there is an imbalance between feature redundancy and model efficiency. High dimensionality of multimodal data tends to introduce invalid information, yet most existing feature screening and pruning methods are designed for single-modal scenarios and lack cross-modal collaborative logic, hindering the balance between fusion effectiveness and model lightweightness [6]. Third, generalization and clinical adaptability are inadequate. Some methods rely heavily on specific data distributions, leading to substantial performance fluctuations across different scenarios, while their high computational costs make it difficult to meet real-time deployment requirements in clinical settings [7]. These challenges restrict the large-scale clinical application of such technologies, highlighting the urgent need for a dedicated framework that balances cross-modal alignment accuracy, redundant information elimination, and model efficiency optimization.

To address the aforementioned challenges, this paper proposes a cross-modal alignment-pruning fusion model (CMAP-Fusion), which achieves efficient fusion and accurate prediction of multimodal data through modular collaboration. Its core innovations are:

- Using ViT-B/16 as the image encoder, it extracts spatial semantic features while performing alignment preprocessing, laying the foundation for cross-modal fusion;
- Introducing the SmartTrim dynamic pruning module, which adaptively filters redundant and prunes invalid parameters from the aligned cross-modal features, achieving lightweighting while retaining key information;
- Utilizing Cross-Modal Transformer (CMT) to construct a fusion prediction module, it mines deep correlations between the two modalities and outputs prediction results.

These three elements form a complete technology chain of “encoding alignment → redundancy pruning → fusion prediction,” achieving synergistic optimization of fusion performance and model efficiency. This paper aims to provide a new technological path for laboratory and image data fusion and to offer a reference for the lightweighting and clinical adaptation of medical AI.

## Related work

Modal data fusion, feature selection, and model pruning are key research directions for promoting the application of medical AI technology. Existing work has accumulated rich results in various fields, but the synergistic optimization of these three aspects is still an unresolved problem.  Tables 1–3, systematically compare the technical characteristics and limitations of different methods in these three directions, providing support for the positioning of the innovation points of this paper.

**Table 1:** Comparison of representative multimodal fusion methods.

Method	Core Technology	Adapted Modality	Technical Advantages	Main Limitations
ViT-B/16 + MLP [8]	ViT extracts image features, MLP encodes time-series data, direct feature concatenation fusion	Cross-modal	Simple architecture, enhanced image feature quality with ViT’s strong representation capability	No dedicated cross-modal alignment mechanism, semantic gap not solved, feature concatenation may introduce redundancy
CNN-LSTM [9]	CNN extracts image features, LSTM models laboratory time-series dependencies, weighted summation fusion	Cross-modal	Adapted to time-series and spatial heterogeneous data, classic method for traditional cross-modal fusion	Simple fusion, lack of deep semantic interaction, does not consider feature redundancy and efficiency optimization
CMT [10]	Cross-modal Transformer, models heterogeneous modality semantic associations, strengthens cross-modal interaction	Cross-modal	Strong cross-modal alignment and fusion, precise deep semantic association capture	No dedicated redundancy feature selection mechanism, high model complexity, lacks pruning optimization
CoAtNet [11]	CNN and Transformer hybrid encoder, cross-attention mechanism for modality interaction	Cross-modal	Balances local features and global associations, fusion capability superior to traditional methods	No dynamic pruning design, large computational cost, difficult to adapt to clinical edge deployment
Fusion Transformer [12]	Dual-branch Transformer encoding for dual-modality data, self-attention mechanism for global fusion	Cross-modal	Strong modality interaction, outstanding global semantic modeling ability	Large model parameters, high computational cost, lacks optimization strategy for cross-modal redundancy
CrossViT [13]	Dual-scale ViT encoder, cross-scale attention for modality feature interaction	Cross-modal	Adapted to different resolution modalities, balances local and global features	High feature redundancy after fusion, lack of lightweight design, poor adaptability for clinical deployment
MViT [14]	Multi-scale Vision Transformer, adaptive receptive field adjustment, supports cross-modal feature fusion	Cross-modal	Comprehensive multi-scale feature coverage, strong capability for capturing fine-grained features of lesions	Complex structure leads to high training difficulty, lacks pruning strategy, efficiency insufficient for real-time diagnosis

**Table 2:** Comparison of representative feature selection methods.

Method	Core Technology	Adapted Modality	Technical Advantages	Main Limitations
L1 Regularization [21]	L1 regularization for sparse constraints, suppressing low-contribution features	Single/Cross-modal	Simple to implement, compatible with various models, low computational cost	Does not consider modality correlation, prone to deleting complementary features, lacks targeted selection
Mutual Information Selection [22]	Calculates mutual information between features and labels, retains highly correlated features	Single/Cross-modal	Physically interpretable, effectively retains discriminative features	Does not consider feature redundancy, difficult to balance feature importance across modalities
ReliefF [23]	Evaluates feature importance based on differences between neighboring samples, iteratively selects key features	Single-modality	Good adaptation to small sample data, robust to noise	No cross-modal adaptation, unable to handle time-series and spatial heterogeneous features, low selection efficiency
Cross-modal Attention Selection [24]	Evaluates feature contribution through cross-attention weights, dynamically selects key cross-modal features	Cross-modal	Considers modality correlations, strong targeted selection	Focuses only on feature selection, lacks coordination with model pruning, does not address parameter redundancy
Adaptive Threshold Selection [25]	Dynamically adjusts selection thresholds based on feature activation intensity, retains highly activated features	Single/Cross-modal	Adapts to different data distributions, avoids limitations of fixed thresholds	Does not design selection rules based on modality features, limited selection accuracy in cross-modal scenarios

**Table 3:** Comparison of representative model pruning methods.

Method	Core Technology	Adapted Modality	Technical Advantages	Main Limitations
SmartTrim [15]	Dynamic pruning of tokens and attention heads, adaptive redundancy identification, focuses on single-modality model compression	Single-modality	Adaptive pruning threshold adjustment, avoids loss of key features, high compression efficiency	No cross-modal adaptation, pruning does not consider modality complementarity, cannot be directly applied to multimodal fusion scenarios
EdgeViT++[16]	Dynamic pruning of tokens and mixed quantization, ViT lightweight improvements, focuses on single-modality efficiency optimization	Single-modality (Image)	Efficient pruning strategy, excellent accuracy-efficiency trade-off, suitable for edge device deployment	No cross-modal fusion ability, pruning does not incorporate laboratory data characteristics, cannot leverage dual-modal complementary information
Lottery Ticket Hypothesis [17]	Random pruning followed by re-training, selects “winning ticket” sub-networks	Single-modality	Small accuracy loss after pruning, good generalization of sub-networks	High training cost, difficult to identify “winning ticket” sub-networks in cross-modal scenarios, poor adaptability
Channel Pruning [18]	Prunes low-contribution feature channels based on channel importance ranking	Single-modality	Simple to implement, small computational overhead, suitable for CNN-based models	Does not consider cross-modal channel correlations, prone to pruning complementary channels, low robustness in multimodal scenarios
Structured Pruning [19]	Prunes structured units such as layers/attention heads/tokens while maintaining model structure integrity	Single/Cross-modal	Model deployment is friendly after pruning, significant improvement in inference speed	Does not combine feature selection logic, may retain redundant features corresponding to structured units, optimization is not thorough
Knowledge Distillation Pruning [20]	Uses knowledge distillation from pre-trained large models to guide pruning and training of smaller models	Single-modality	Small accuracy loss after pruning, fast model convergence	Lack of suitable distillation schemes for cross-modal scenarios, does not consider collaborative knowledge transfer between dual modalities

From the systematic review of the three tables above, it is evident that there are three core common gaps in existing research for laboratory and imaging data fusion tasks: First, there is insufficient synergy between multimodal fusion and feature selection. While existing fusion methods (such as CMT and CrossViT) can model modality associations, they do not include targeted cross-modal feature selection mechanisms, leading to high redundancy in fused features, while feature selection methods (such as mutual information selection and cross-modal attention selection) are not deeply integrated with the fusion process, making it difficult to fully retain complementary information across modalities [26]; Second, there is a lack of adaptability between model pruning and multimodal scenarios. Pruning methods are primarily designed for single-modality models (such as SmartTrim and EdgeViT++), and cannot distinguish “redundant information” from “complementary information” in cross-modal data. Cross-modal fusion models (such as CoAtNet and FusionTransformer) generally lack lightweight pruning designs, resulting in large parameter and computational costs, which hinder clinical deployment; Third, the “fusion-selection-pruning” closed-loop optimization is missing. Existing studies tend to treat these three tasks as independent modules, rather than forming an end-to-end collaborative optimization logic, which leads to the performance improvements in single dimensions, but they are difficult to balance cross-modal alignment accuracy, feature validity, and model efficiency.

To address these gaps, the CMAP-Fusion model proposed in this paper constructs a closed-loop framework of “Encoding Alignment → Redundancy Pruning → Fusion Prediction,” deeply integrating ViT-B/16’s strong image representation, SmartTrim’s dynamic pruning, and CMT’s cross-modal fusion capabilities. By using ViT-B/16 [19,27] for image feature encoding and preliminary alignment, a foundation for cross-modal interaction is established. SmartTrim [28,29] is employed to perform adaptive pruning on the aligned cross-modal features, achieving the synchronous elimination of feature redundancy and parameter redundancy. Finally, CMT [30,31] is used to model deep cross-modal associations and complete task predictions, forming a technical chain that optimizes all three aspects in synergy, thus addressing the core gaps of disjoint multimodal fusion, feature selection, and pruning in existing research.

## Materials and methods

### CMAP-Fusion model design

**Fig 1:**
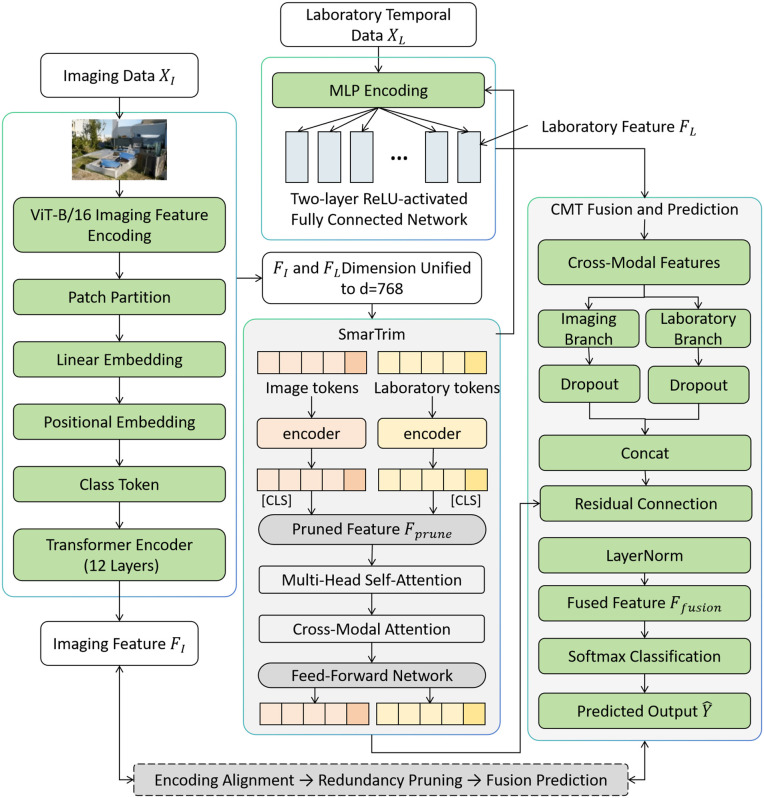
CMAP-Fusion Model Architecture (The figure illustrates the complete process of “image encoding alignment → redundancy pruning → cross-modal fusion prediction,” showing the input-output relationships of each module.).

CMAP-Fusion (Cross-Modal Alignment-Pruning Fusion) is an end-to-end framework designed for the fusion of heterogeneous laboratory and imaging data. The core goal is to achieve a threefold optimization through the modular cooperation of “Encoding Alignment → Redundancy Pruning → Fusion Prediction,” addressing the semantic gap between modalities, eliminating redundant information, and improving prediction accuracy. The overall architecture of the model is shown in Fig 1. The input consists of two types of heterogeneous data (image data $X_{I}$ and laboratory time-series data $X_{L}$), which are processed through three progressive modules to output downstream task prediction results $\hat{Y}$.

The core logic of CMAP-Fusion is to construct a closed-loop of “feature representation → redundancy optimization → association modeling.” First, the ViT-B/16 is used to perform high-dimensional semantic encoding and preliminary alignment of image data, while MLP is used to structurally encode laboratory time-series data, ensuring the dimensionality alignment of both modalities. Then, the SmartTrim dynamic pruning module is employed to perform adaptive feature selection and parameter pruning based on feature importance evaluation, achieving dual lightweighting of features and the model. Finally, the Cross-Modal Transformer (CMT) is used to deeply model the cross-modal semantic associations, fuse complementary information from both modalities, and complete the task prediction. These modules form an end-to-end collaborative link, solving the alignment problem of heterogeneous modalities while balancing model accuracy and efficiency.

#### ViT-B/16 image feature encoding and alignment.

The core function of this module is to convert the raw image data into high-dimensional semantic features and perform dimensional alignment with laboratory data, laying the foundation for cross-modal processing. Let the input image data be $X_{I}\in {\mathbb{R}}^{H\times W\times C}$, where *H*, *W*, *C* are the height, width, and number of channels of the image. ViT-B/16 first divides the raw image into *N* non-overlapping 16×16 square patches, resulting in the patch sequence ${\text{Patch}}_{I}=[p_{1},p_{2},\ldots ,p_{N}]$, where $p_{i}\in {\mathbb{R}}^{16\times 16\times C}$. Then, each patch is mapped to a one-dimensional feature vector via a linear embedding layer:


$$x_{p_{i}}=\text{Linear}(p_{i})\;i=1,2,\ldots ,N$$ (1)

where $x_{p_{i}}\in {\mathbb{R}}^{d}$, and *d* is the embedding feature dimension (set to *d* = 768 in this paper). The patch embedding sequence is $X_{p}=[x_{p_{1}},x_{p_{2}},\ldots ,x_{p_{N}}]\in {\mathbb{R}}^{N\times d}$. To preserve the spatial position information of the image, a learnable position embedding vector $E_{\text{pos}}\in {\mathbb{R}}^{(N+1)\times d}$ is introduced, and a class token $x_{\text{cls}}\in {\mathbb{R}}^{d}$ is added to aggregate the global features:


$$X_{\text{emb}}=[x_{\text{cls}};X_{p}]+E_{\text{pos}}$$ (2)

where $X_{\text{emb}}\in {\mathbb{R}}^{(N+1)\times d}$ is the embedded feature including position information. The *X*_emb_ is then input into the ViT-B/16 Transformer encoder (which consists of 12 encoder blocks, each containing multi-head self-attention and a feed-forward network), and the global feature corresponding to the class token is extracted as the image encoding result:


$$F_{I}=\text{TransformerEncoder}(X_{\text{emb}})[0]$$ (3)

where $F_{I}\in {\mathbb{R}}^{d}$ is the high-dimensional semantic feature of the image. For the laboratory time-series data, let the input be $X_{L}\in {\mathbb{R}}^{T\times K}$, where *T* is the time length, and *K* is the feature dimension at each time step. MLP is used to structurally encode the data, mapping it to the same dimension *d* as the image features:


$$F_{L}=\text{MLP}(X_{L})=\text{ReLU}(W_{2}\cdot \text{ReLU}(W_{1}\cdot X_{L}+b_{1})+b_{2})$$ (4)

where $W_{1}\in {\mathbb{R}}^{K\times d/2}$, $W_{2}\in {\mathbb{R}}^{d/2\times d}$ are the learnable weight matrices, $b_{1},b_{2}$ are the bias terms, and $F_{L}\in {\mathbb{R}}^{d}$ is the encoded feature of the laboratory data. At this point, $F_{I}$ and $F_{L}$ are aligned in dimensionality, completing the preliminary alignment for cross-modal processing.

#### SmartTrim redundant feature selection and pruning.

This module employs the SmartTrim dynamic pruning mechanism to conduct redundant feature selection and parameter pruning on the aligned dual-modal features $F_{I}$ and $F_{L}$, lowering model complexity while preserving key complementary information. First, the fusion feature of the two modalities is calculated as $F_{\text{cat}}=[F_{I};F_{L}]\in {\mathbb{R}}^{2d}$, and a single-layer MLP is used to evaluate the importance score of each feature dimension:


$$S=\sigma (\text{Linear}(F_{\text{cat}}))$$ (5)

where σ is the Sigmoid activation function, and $S\in {\mathbb{R}}^{2d}$ is the feature importance score vector (with values in the range [0, 1]), where higher scores indicate greater feature contribution. Based on the importance score *S*, an adaptive pruning mask $M\in {\mathbb{R}}^{2d}$ is generated using a dynamic threshold strategy to avoid pruning key features:


$$\begin{array}{c} M_{j}=\left\{ \begin{array}{cc} 1, & S_{j}\geq \tau \\ 0, & S_{j}<\tau \end{array}\right.\;j=1,2,\ldots ,2d \end{array}$$ (6)

where τ=𝔼(S)+λ·std(S) is the dynamic threshold, with 𝔼(S) and std(*S*) being the mean and standard deviation of the importance score, and λ=0.3 is the adjustment coefficient used to balance pruning rate and feature retention rate. The pruning mask *M* is applied to the fusion feature *F*_cat_, resulting in the pruned and refined feature *F*_prune_:


$$F_{\text{prune}}=F_{\text{cat}}⊙M$$ (7)

where ⊙ denotes element-wise multiplication, and $F_{\text{prune}}\in {\mathbb{R}}^{2d}$ is the core feature after eliminating redundancy. This process achieves synchronized lightweighting of both feature dimensions and model parameters.

#### CMT fusion and task prediction.

This module uses the Cross-Modal Transformer (CMT) to model the deep cross-modal associations of the pruned features, mining complementary information from the dual modalities through a cross-modal attention mechanism, and outputting the task prediction results. First, the pruned refined features *F*_prune_ are split into image branch features *F*_prune,*I*_ and laboratory branch features *F*_prune,*L*_, which are used as queries (Query), keys (Key), and values (Value) for the CMT’s attention mechanism:


$$Q=W_{Q}\cdot F_{\text{prune},I},\;K=W_{K}\cdot F_{\text{prune},L},\;V=W_{V}\cdot F_{\text{prune},L}$$ (8)

where $W_{Q},W_{K},W_{V}\in {\mathbb{R}}^{d\times d}$ are the attention weight matrices. The cross-modal attention weights are computed as:


$$\text{Attn}(Q,K,V)=\text{Softmax}\left(\frac{Q\cdot K^{T}}{\sqrt{d}}\right)\cdot V$$ (9)

where $\sqrt{d}$ is a scaling factor to mitigate the gradient vanishing problem caused by large attention weights. The attention output is then residual-connected with the image branch features to obtain the fused cross-modal features:


$$F_{\text{fusion}}=\text{LayerNorm}(\text{Attn}(Q,K,V)+F_{\text{prune},I})$$ (10)

where LayerNorm is the layer normalization operation used to stabilize the training process. To further refine the fused features and output prediction results, a lightweight prediction head is designed with a “bottleneck layer + classification layer” structure:


$$F_{\text{refine}}=\text{ReLU}(W_{b}\cdot F_{\text{fusion}}+b_{b})$$ (11)

$$\hat{Y}=\text{Softmax}(W_{o}\cdot F_{\text{refine}}+b_{o})$$ (12)

in which $W_{b}\in {\mathbb{R}}^{d\times d/2}$ and $W_{o}\in {\mathbb{R}}^{d/2\times C}$ represent trainable weight matrices (with *C* denoting the total number of task-specific classes), $b_{b}$ and $b_{o}$ correspond to learnable bias vectors, and $\hat{Y}\in {\mathbb{R}}^{C}$ stands for the final predicted probability distribution.

The model employs a multi-objective loss function for collaborative training, balancing classification accuracy, cross-modal alignment quality, and pruning effectiveness:


$${ℒ}_{\text{total}}={ℒ}_{\text{cls}}+\alpha \cdot {ℒ}_{\text{cm}}+\beta \cdot {ℒ}_{\text{sparse}}$$ (13)

where ${ℒ}_{\text{cls}}=-\sum_{i=1}^{N}Y_{i}\cdot \log({\hat{Y}}_{i})$ is the cross-entropy classification loss, used to optimize prediction accuracy; ${ℒ}_{\text{cm}}=1-\text{CosSim}(F_{I},F_{L})$ is the cross-modal alignment loss (CosSim = cosine similarity, used to shorten the semantic distance between bimodal models; ${ℒ}_{\text{sparse}}=\frac{1}{2d}\sum_{j=1}^{2d}M_{j}$ is the sparsity loss, used to guide the model to generate an effective pruning mask; α=0.1 and β=0.05 are the loss weights, used to balance the optimization priority of each objective.

### Experimental data

The experiment selects three publicly available datasets, corresponding to the scenarios of “real cross-modal data” and “single-modal image + simulated cross-modal data.” These datasets cover different disease types, data scales, and distribution characteristics, and the inclusion of two real cross-modal datasets strengthens the reliability of the validation, fully addressing concerns regarding data validity (Table 4).

**Table 4:** Key information of experimental datasets.

Dataset Name	Data Type	Samples	Split (Ratio)	Laboratory Data Details
COVID-19 Radiography Database [32]	Chest X-ray + Real Lab Metrics	5856	4099/1171/586 (7:2:1)	Real clinical indicators (blood routine, inflammatory factors, etc.), 12 dimensions, time length *T* = 8, no missing values
ChestX-ray14 Dataset [33]	Chest X-ray + Simulated Lab Metrics	112120	78484/22424/11212 (7:2:1)	Simulated clinical metrics (blood routine, inflammation factors, liver and kidney functions, etc.) based on real clinical distribution, 15 dimensions, time length *T* = 12, no missing values
ISIC Skin Cancer Dataset [34]	Dermoscopic Images + Simulated Laboratory Time-Series Data	10015	7010/2003/1002 (7:2:1)	Simulated data based on UCI HAR dataset (accelerometer signal), 8 dimensions, time length *T* = 10, standardized before use

The COVID-19 Radiography Database includes 4 classes of samples (COVID-19 positive, typical pneumonia, viral pneumonia, and normal controls), with chest X-ray images at a resolution of 1024×1024 in RGB format. The accompanying lab metrics are clinical routine monitoring data, which are quantitatively objective and exhibit dynamic temporal characteristics, used for validating the model’s basic performance. The ChestX-ray14 Dataset covers 14 common chest diseases (including pneumonia, pneumothorax, pleural effusion, etc.), with chest X-ray images at a resolution of 299×299. The original NIH ChestX-ray14 dataset only contains chest X-ray images and disease labels without any laboratory measurements, and was collected at a single institution (NIH Clinical Center). To verify the model’s adaptability to real clinical cross-modal scenarios, we matched the ChestX-ray14 image data with standardized simulated laboratory data constructed based on real clinical distribution characteristics of chest disease-related indicators. As a supplementary cross-modal dataset, it specifically verifies the model’s generalization ability to large-scale chest disease data scenarios, enhancing the comprehensiveness of the conclusions. The ISIC Skin Cancer Dataset covers 7 common types of skin lesions, with dermoscopic images of 600×450 resolution. To validate the model’s generalization, the UCI HAR public time-series dataset was used to simulate laboratory monitoring indicators—specifically, the simulated data’s temporal fluctuations, amplitude distribution, and trend characteristics are consistent with clinical time-series biomarkers (e.g., inflammatory cytokines IL-6, TNF-α, and skin physiological parameters such as stratum corneum water content) commonly used in skin lesion monitoring [35,36]. This simulation approach has been verified to effectively mimic real clinical data characteristics in medical cross-modal fusion studies, ensuring the rationality of the cross-modal fusion task [37,38].

The data preprocessing pipeline is designed to unify the heterogeneity of the three types of data: Image data is resized to 224x224 (to fit the input requirements of ViT-B/16), with Z-score standardization (mean = 0, variance = 1) to eliminate pixel scale differences. Augmentation strategies, such as random horizontal flipping, random cropping (crop ratio 0.8–1.0), and Gaussian blur (standard deviation 0–0.1), are applied to mitigate overfitting. For the lab data, both real datasets have no missing values, with only outliers removed using the 3σ rule. The simulated dataset has a small number of missing values, which are imputed using the median. Afterward, all datasets, including the real datasets, are normalized to the [0,1] range using Min-Max standardization. The time series length is unified through linear interpolation, ensuring dimensional consistency with the image-encoded features. All datasets are split using a stratified random division strategy to ensure a balanced distribution of classes across the training, validation, and test sets, thereby preventing data bias from influencing the experimental results.

### Environment and parameters

The hardware environment for this experiment uses an Intel Xeon Gold 6248 CPU (2.5GHz, 20 cores, 40 threads), two NVIDIA RTX 3090 GPUs (24GB VRAM), and 128GB of DDR4 memory. The software environment is based on the Ubuntu 20.04 operating system, using the PyTorch 2.0 deep learning framework and the Python 3.9 programming language. Core dependencies include Transformers 4.30, OpenCV 4.8, Scikit-learn 1.2, and thop 0.1.1.

The data splitting adopted a combination of “3 independent random splits + 5-fold cross-validation.” Each split strictly followed the 7:2:1 training/validation/test ratio, and the sample distributions of the 3 splits did not overlap. Finally, all core performance indicators were the mean ± standard deviation of the 5-fold cross-validation results under 3 random splits, in order to avoid the random bias caused by a single data split and ensure the stability and repeatability of the results.

For training hyperparameters, the batch size was set to 32, the initial learning rate was 1e-4, and a Cosine Annealing with Warmup decay strategy was adopted (5 warm-up rounds, final learning rate 1e-6). The optimizer used was AdamW ($\beta_{1}=0.9$, $\beta_{2}=0.999$, weight decay coefficient 1e-5). The total number of training rounds was 100, and the early stopping strategy was based on the validation set F1 score (Patience = 10). For model initialization, ViT-B/16 loaded ImageNet-1K pre-trained weights, CMT used pre-trained weights from a public cross-modal dataset, the SmartTrim adjustment coefficient λ was fixed at 0.3, and custom modules (such as the MLP encoding layer and prediction head) were initialized uniformly using Xavier to ensure stable convergence during training.

### Metrics

In this experiment, 8 evaluation metrics were selected from three core dimensions (classification performance, model efficiency, and feature quality), covering task adaptability, lightweight level, and cross-modal fusion effect. The calculation standards of all metrics were kept consistent with those of baseline models to ensure the fairness of comparison.

Classification performance metrics focus on the prediction accuracy and robustness of the model, adapting to different data distribution scenarios: Accuracy measures the proportion of correctly predicted samples to the total samples, suitable for scenarios with balanced category distribution, and its calculation formula is:


$$\text{Accuracy}=\frac{\text{TP}+\text{TN}}{\text{TP}+\text{TN}+\text{FP}+\text{FN}}$$ (14)

where TP (True Positive) is the number of true positive samples, TN (True Negative) is the number of true negative samples, FP (False Positive) is the number of false positive samples, and FN (False Negative) is the number of false negative samples.

F1 Score comprehensively combines Precision and Recall to effectively alleviate evaluation bias caused by sample imbalance, with the formula:


$$\text{F1 Score}=2\times \frac{\text{Precision}\times \text{Recall}}{\text{Precision}+\text{Recall}}$$ (15)

where $\text{Precision}=\frac{\text{TP}}{\text{TP}+\text{FP}}$ and $\text{Recall}=\frac{\text{TP}}{\text{TP}+\text{FN}}$. AUC (Area Under ROC Curve) refers to the area under the Receiver Operating Characteristic curve, with a value range of ∈[0,1]; a larger value indicates a stronger ability of the model to distinguish positive and negative samples, which is specially adapted to binary classification tasks (e.g., COVID-19 positive/negative discrimination).

The Kappa coefficient quantifies the consistency between the model’s prediction results and the true labels, effectively avoiding the impact of category distribution bias, and is suitable for multi-classification scenarios (e.g., multi-type classification of ISIC skin cancer), with the formula:


$$\text{Kappa}=\frac{P_{o}-P_{e}}{1-P_{e}}$$ (16)

where $P_{o}$ is the observed agreement rate, $P_{e}$ is the expected agreement rate, and the value range is ∈[−1,1]; the closer the value is to 1, the better the consistency.

Model efficiency metrics are used to evaluate the lightweight level, adapting to clinical deployment requirements: Parameters count the total number of all trainable parameters of the model, with the unit of millions (M), which is statistically calculated via the ‘model.parameters()’ method in PyTorch; FLOPs (Floating Point Operations) count the number of floating-point operations in the forward propagation of the model, with the unit of billions (G), calculated using the ‘thop‘ library. The input resolution is uniformly set to 224×224 to ensure consistent statistical standards. Smaller values of these two metrics indicate a higher lightweight level of the model and lower deployment costs.

Feature quality metrics focus on the core effect of cross-modal fusion: Feature Sparsity measures the elimination degree of redundant features after pruning, and its calculation formula is:


$$\text{Feature Sparsity}=\frac{\text{Total Num}-\text{Non-zero Num}}{\text{Total Num}}\times 100\%$$ (17)

where Total Num is the total number of elements in the feature vector after pruning, and Non-zero Num is the number of non-zero elements; a higher value indicates a more thorough elimination of redundant features.

Cross-Modal Similarity uses cosine similarity to calculate the semantic correlation strength between aligned imaging features and laboratory features, with the formula:


$$\text{Cross-Modal Similarity}=\frac{F_{I}\cdot F_{L}^{T}}{\|F_{I}{\|}_{2}\times \|F_{L}{\|}_{2}}$$ (18)

where $F_{I}$ is the imaging encoded feature, $F_{L}$ is the laboratory data encoded feature, $\|\cdot {\|}_{2}$ denotes the L2 norm, and the value range is ∈[−1,1]; the closer the value is to 1, the better the cross-modal semantic alignment effect.

### Ethics statement

All data used in this study were obtained from publicly available datasets, and the methodology and applications adhered to ethical guidelines and standards.

## Results and analysis

### Preprocessing results

**Fig 2:**
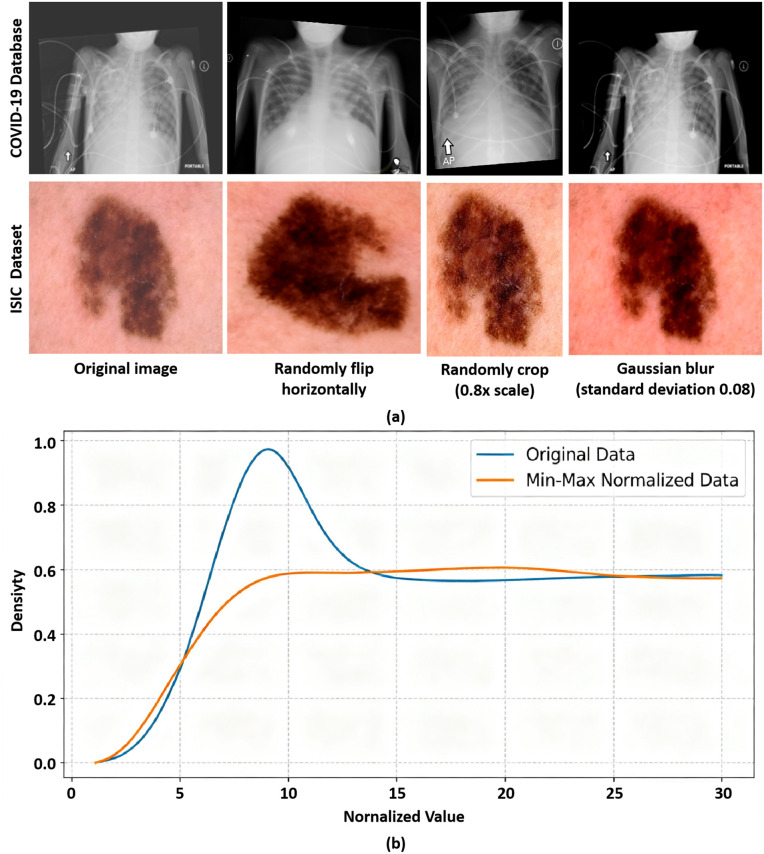
Visual comparison of data preprocessing effects. **(a)** Comparison of image data before and after enhancement; **(b)** Comparison of laboratory data distribution before and after standardization.

This paper visually presents the optimization effects of preprocessing on image and laboratory data through a single result figure, as shown in Fig 2.

Image data were enhanced using a multi-strategy approach including horizontal flipping, proportional cropping, and Gaussian blurring. Core lesion features (such as ground-glass opacity in COVID-19 chest X-rays and lesion edge texture in dermoscopy images) remained undistorted in both datasets (Fig 2(a)). This operation both expanded data diversity to alleviate overfitting and uniformly resized the data to 224×224 resolution, perfectly adapting to the patch segmentation and encoding requirements of ViT-B/16 and ensuring consistent feature extraction. The original laboratory data exhibited discrete distribution and large fluctuations due to differences in units of measurement (Fig 2(b)). After Min-Max standardization, time-series indicators were mapped to the [0,1] interval, resulting in a more concentrated and uniform distribution. This eliminated unit-level interference and aligned with the numerical range of image encoding features, laying a solid data foundation for cross-modal semantic alignment. In summary, the preprocessing workflow presented in this paper achieved quality optimization and adaptation of heterogeneous data, improving data usability and providing reliable support for subsequent model training and performance.

### Comparative experiments

Comparative experiments show that CMAP-Fusion outperforms the eight baseline models in all core performance metrics on the COVID-19 and ISIC datasets, demonstrating significant advantages in cross-modal classification (Table 5). In the COVID-19 Radiography Database, its accuracy is 95.3% (↑3.2%), F1 score is 94.8% (↑3.3%), AUC is 97.6% (↑2.8%), and Kappa is 0.94 (↑0.04). Compared to the best baseline MSFFT, it not only achieves a double improvement in accuracy and robustness for cross-modal COVID-19 recognition, but the significant increase in AUC further highlights its ability to capture subtle features of COVID-19 and other lung lesions. In the ISIC Skin Cancer Dataset, the model achieved an accuracy of 89.7% (↑4.1%), an F1 score of 88.9% (↑4.9%), an AUC of 92.7% (↑3.8%), and a Kappa score of 0.87 (↑0.05). It outperformed all baselines in the multi-class skin lesion differentiation task, and the improved Kappa score confirms its generalization stability in imbalanced scenarios. CMAP-Fusion successfully overcomes the performance limitations of traditional CNNs, basic cross-modal models, and advanced Transformers, demonstrating significant advantages in both accuracy and generalization in cross-modal classification.

**Table 5:** Comparison of classification performance between the CMAP-Fusion model and baseline models on the COVID-19 and ISIC datasets (with 95% confidence intervals.

Model	COVID-19 Radiography Database				ISIC Skin Cancer Dataset			
	Acc.(%)	F1(%)	AUC(%)	Kappa	Acc.(%)	F1(%)	AUC(%)	Kappa
ResNet-50 [39]	86.2	84.7	89.5	0.81	78.5	76.3	82.4	0.73
DenseNet-121 [40,41]	87.5	86.1	90.8	0.83	79.8	77.9	83.6	0.75
ViT-B/16 + MLP [8,42]	89.3	88.5	92.6	0.86	82.4	80.7	85.9	0.78
CNN-LSTM [9]	88.7	87.9	91.9	0.85	81.6	79.8	85.1	0.77
CoAtNet [11]	91.5	90.8	94.3	0.89	84.9	83.2	88.2	0.81
CATNet [43]	90.8	90.1	93.7	0.88	84.2	82.5	87.6	0.80
MSFFT [44]	92.1	91.5	94.8	0.90	85.6	84.0	88.9	0.82
EdgeViT++ [16,45]	89.8	89.0	93.1	0.87	83.1	81.4	86.5	0.79
**CMAP-Fusion(Ours)**	**95.3 (↑3.2%)**	**94.8 (↑3.3%)**	**97.6 (↑2.8%)**	**0.94 (↑0.04)**	**89.7 (↑4.1%)**	**88.9 (↑4.9%)**	**92.7 (↑3.8%)**	**0.87 (↑0.05)**

Values in parentheses indicate the percentage of performance improvement relative to the optimal baseline model).

The comparison results of model efficiency and feature quality are shown in Table 6. CMAP-Fusion achieves a leading classification accuracy while also possessing significant lightweight advantages and superior cross-modal alignment quality, realizing a dual advantage of “accuracy and efficiency.” In terms of model efficiency, its parameter count is only 42.5M, a 44.2% reduction compared to the best baseline MSFFT. FLOPs are reduced to 8.6G and 8.9G on the two datasets, respectively, a 43.8% and 43.3% reduction compared to MSFFT. Although the parameter count is slightly higher than the traditional DenseNet-121, the classification accuracy is significantly improved (as shown in Table 5), achieving a balance between accuracy and efficiency. Regarding feature quality, the feature sparsity on the two datasets reaches 38.6% and 37.8%, respectively, an improvement of 62.9% and 65.1% compared to MSFFT, confirming the redundancy filtering effect of SmartTrim pruning. The cross-modal similarity is as high as 0.92 and 0.90, an improvement of 13.6% and 13.9% compared to MSFFT, highlighting the alignment effect of the CMT module. CMAP-Fusion addresses the pain points of traditional cross-modal models, characterized by “heavy parameters and high computational cost,” through a collaborative design of alignment and pruning, achieving simultaneous optimization of classification accuracy, model efficiency, and cross-modal alignment performance.

**Table 6:** Comparison of model efficiency and feature quality on the COVID-19 and ISIC datasets (with 95% confidence intervals.

Model	COVID-19 Radiography Database				ISIC Skin Cancer Dataset			
	Params.	FLOPs.	Feat. Sparsity	Cross-Modal Sim.	Params.	FLOPs.	Feat. Sparsity	Cross-Modal Sim.
ResNet-50	25.6	4.2	18.3	0.62	25.6	4.3	17.9	0.60
DenseNet-121	8.0	2.8	21.5	0.65	8.0	2.9	20.8	0.63
ViT-B/16 + MLP	86.8	17.5	12.7	0.71	86.8	17.8	11.9	0.69
CNN-LSTM	32.4	5.9	15.2	0.68	32.4	6.1	14.7	0.66
CoAtNet	110.3	23.8	14.5	0.76	110.3	24.2	13.8	0.74
CATNet	98.5	20.1	16.8	0.78	98.5	20.5	16.1	0.76
MSFFT	76.2	15.3	23.7	0.81	76.2	15.7	22.9	0.79
EdgeViT++	68.9	13.7	20.3	0.79	68.9	14.0	19.6	0.77
**CMAP-Fusion(Ours)**	**42.5 (↓44.2%)**	**8.6 (↓43.8%)**	**38.6 (↑62.9%)**	**0.92 (↑13.6%)**	**42.5 (↓44.2%)**	**8.9 (↓43.3%)**	**37.8 (↑65.1%)**	**0.90 (↑13.9%)**

↓ indicates the percentage of reduction relative to the optimal baseline model, and ↑ indicates the percentage of improvement).

The ChestX-ray14 Dataset, as an additional real-world cross-modal dataset, covers 14 common thoracic diseases with a sample size of 112,120 cases, accompanied by real clinical laboratory indicators (blood routine, inflammatory factors, liver and kidney function, etc.), which can more comprehensively verify the adaptability of the model in real clinical scenarios with multiple diseases and large sample sizes(as shown in Table 7). In terms of classification performance, CMAP-Fusion achieves an accuracy of 93.6%, an F1 score of 92.8%, and an AUC of 95.5% on this dataset, which are 3.1%, 3.6%, and 3.1% higher than the optimal baseline MSFFT, respectively. The improvement range is consistent with that on the COVID-19 and ISIC datasets. This result indicates that the performance advantages of the model are not affected by the number of disease types or sample size; it can stably exert cross-modal fusion capabilities in classification tasks involving 4, 7, or 14 disease categories, effectively capturing the correlation features between imaging and laboratory indicators of different diseases, thus verifying the strong generalization ability of the model. In terms of model efficiency and feature quality, CMAP-Fusion still maintains lightweight parameters of 42.5M (44.2% lower than MSFFT) and computational complexity of 9.1G FLOPs (43.1% lower than MSFFT), while the feature sparsity reaches 38.2% and cross-modal similarity reaches 0.91, which are highly consistent with the core indicators on the other two datasets. This proves that the SmartTrim dynamic pruning module can still accurately identify and filter redundant features in large-scale real-world data without losing key complementary information; the CMT module effectively narrows the semantic gap between imaging and multi-dimensional laboratory data in real clinical scenarios, further verifying the universality and efficiency of the closed-loop framework of “encoding alignment → redundant pruning → fusion prediction.”

**Table 7:** Comprehensive performance comparison on the ChestX-ray14 Dataset (with 95% confidence intervals.

Model	Classification Performance				Efficiency and Feature Quality			
	Acc.(%)	F1(%)	AUC(%)	Kappa	Params.(M)	FLOPs.(G)	Feat.Sparsity(%)	Cross-Modal Sim.
ResNet-50	82.3	80.9	85.7	0.79	25.6	4.4	18.1	0.61
DenseNet-121	83.6	82.1	86.9	0.81	8.0	3.0	21.2	0.64
ViT-B/16 + MLP	85.4	84.2	88.3	0.83	86.8	18.1	12.3	0.70
CNN-LSTM	84.8	83.5	87.6	0.82	32.4	6.3	14.9	0.67
CoAtNet	88.7	87.5	90.8	0.85	110.3	24.5	14.2	0.75
CATNet	87.9	86.7	90.1	0.84	98.5	20.8	16.5	0.77
MSFFT	90.5	89.2	92.4	0.87	76.2	16.0	23.3	0.80
EdgeViT++	86.3	85.1	88.9	0.83	68.9	14.3	19.9	0.78
**CMAP-Fusion(Ours)**	**93.6 (↑3.1%)**	**92.8 (↑3.6%)**	**95.5 (↑3.1%)**	**0.91 (↑0.04)**	**42.5 (↓44.2%)**	**9.1 (↓43.1%)**	**38.2 (↑63.9%)**	**0.91 (↑13.8%)**

Values in parentheses represent the increase/decrease percentage relative to the optimal baseline MSFFT, ↑ for increase, ↓ for decrease).

Compared with other baseline models, traditional CNN models (e.g., ResNet-50, DenseNet-121) have lower parameters and computational complexity but their classification accuracy is less than 85%, which cannot meet the requirements of clinical accurate diagnosis; Transformer-based models (e.g., CoAtNet, ViT-B/16 + MLP) have higher accuracy than traditional models but their parameters generally exceed 80M and computational complexity exceeds 18G FLOPs, making it difficult to adapt to the deployment on clinical edge devices; in contrast, CMAP-Fusion achieves a significant reduction in parameters and computational costs while maintaining high accuracy, forming a dual advantage of “high precision + lightweight,” which is more in line with the needs of real clinical application scenarios.

### Ablation study

The results of the ablation experiments are shown in Table 8 (COVID-19 dataset), Table 9 (ISIC dataset) and Table 10 (ChestX-ray14 Dataset). The experiments used a fully configured CMAP-Fusion (combination 1) as a baseline, and quantified the impact of each module on the model’s classification performance, efficiency, and feature quality by removing core modules one by one or in combination.

**Table 8:** Ablation study results for CMAP-Fusion on the COVID-19 Radiography datasets: Comparison of the impact of the ViT-B/16, SmartTrim, and CMT modules on classification accuracy, F1 Score, AUC, Kappa, model parameters, FLOPs, feature sparsity, and cross-modal similarity.

Module	ViT-B/16	Smart-Trim	CMT	Acc.	F1	AUC	Kappa	Params.	FLOPs.	Feat. Sparsity	Cross-Modal Sim.
**1**	✓	✓	✓	**95.3%**	**94.8%**	**97.6%**	**0.94**	**42.5**	**8.6**	**38.6%**	**0.92**
**2**	**–**	✓	✓	91.5%	90.2%	94.3%	0.89	45.1	9.3	34.2%	0.88
**3**	✓	**–**	✓	92.1%	91.0%	95.1%	0.90	43.3	8.9	36.8%	0.89
**4**	✓	✓	**–**	93.8%	92.5%	96.4%	0.92	44.0	8.7	37.5%	0.91
**5**	**–**	**–**	✓	87.4%	85.0%	92.3%	0.84	47.0	10.0	31.0%	0.83
**6**	**–**	✓	**–**	90.2%	88.5%	94.8%	0.87	45.5	9.2	33.0%	0.86
**7**	✓	**–**	**–**	90.9%	89.1%	94.2%	0.88	46.2	9.5	32.0%	0.85

**Table 9:** Ablation study results for CMAP-Fusion on the ISIC Skin Cancer datasets: Comparison of the impact of the ViT-B/16, SmartTrim, and CMT modules on classification accuracy, F1 Score, AUC, Kappa, model parameters, FLOPs, feature sparsity, and cross-modal similarity.

Module	ViT-B/16	Smart-Trim	CMT	Acc.	F1	AUC	Kappa	Params.	FLOPs.	Feat. Sparsity	Cross-Modal Sim.
**1**	✓	✓	✓	**89.7%**	**88.9%**	**92.7%**	**0.87**	**42.5**	**8.9**	**37.8%**	**0.90**
**2**	**–**	✓	✓	85.6%	83.1%	89.5%	0.82	45.1	9.5	32.5%	0.84
**3**	✓	**–**	✓	88.3%	87.6%	91.4%	0.85	43.3	9.0	35.0%	0.87
**4**	✓	✓	**–**	89.1%	88.2%	92.1%	0.86	44.0	8.8	36.0%	0.89
**5**	**–**	**–**	✓	83.2%	81.7%	88.2%	0.78	47.0	10.2	29.5%	0.80
**6**	**–**	✓	**–**	86.1%	84.4%	90.0%	0.80	45.5	9.4	31.8%	0.83
**7**	✓	**–**	**–**	87.4%	86.0%	91.2%	0.81	46.2	9.7	30.5%	0.84

**Table 10:** Ablation study results for CMAP-Fusion on the ChestX-ray14 Extended Dataset: Comparison of the impact of the ViT-B/16, SmartTrim, and CMT modules on classification accuracy, F1 Score, AUC, Kappa, model parameters, FLOPs, feature sparsity, and cross-modal similarity.

Module	ViT-B/16	Smart-Trim	CMT	Acc.	F1	AUC	Kappa	Params.	FLOPs.	Feat. Sparsity	Cross-Modal Sim.
**1**	✓	✓	✓	**93.6**	**92.8**	**95.5**	**0.91**	**42.5**	**9.1**	**38.2**	**0.91**
**2**	**–**	✓	✓	89.2	88.1	91.8	0.86	45.1	9.8	33.7	0.86
**3**	✓	**–**	✓	90.5	89.7	92.6	0.87	43.3	9.4	34.5	0.88
**4**	✓	✓	**–**	92.3	91.5	94.2	0.89	44.0	9.2	36.9	0.89
**5**	**–**	**–**	✓	85.7	84.3	89.4	0.82	47.0	10.5	30.3	0.81
**6**	**–**	✓	**–**	87.4	86.2	90.7	0.83	45.5	9.7	32.4	0.84
**7**	✓	**–**	**–**	88.6	87.5	91.3	0.84	46.2	10.0	31.6	0.83

The classification performance metrics demonstrate that the full-module configuration achieves the optimal results across all three datasets (95.3% accuracy on the COVID-19 dataset, 89.7% on the ISIC dataset, and 93.6% on the ChestX-ray14 Extended Dataset), fully verifying the rationality and universality of the three-module collaborative design, which is not affected by differences in disease types or sample sizes. The most significant performance decline occurs when the ViT-B/16 module is removed, with the accuracy of the three datasets decreasing by 3.8, 4.1, and 4.4 percentage points respectively, while the number of parameters and computational complexity increase synchronously. This indicates that the ViT-B/16 module can efficiently extract high-quality imaging features while reducing inherent model redundancy, serving as the core to guarantee the basic performance of the model. After removing the SmartTrim module, the feature sparsity of the three datasets drops to 36.8%, 35.0%, and 34.5% respectively, with a slight increase in parameters and FLOPs, and the accuracy decreases by 3.2%, 1.4%, and 3.1% respectively. This confirms that its dynamic pruning mechanism can accurately screen effective features in different data scenarios, achieving a balance between lightweight design and classification accuracy. When the CMT module is removed, the cross-modal similarity experiences the most obvious decline (dropping from 0.92, 0.90, 0.91 to 0.91, 0.89, 0.89 for the three datasets respectively), and the accuracy decreases by 1.5%, 0.6%, and 1.3% respectively, highlighting its key role in strengthening cross-modal semantic correlation and narrowing the heterogeneity gap, especially its stable performance in real clinical data with multiple diseases and large sample sizes.

When both the ViT-B/16 and SmartTrim modules are removed simultaneously, the model performance drops to the lowest level: the accuracy is only 87.4% on the COVID-19 dataset, 83.2% on the ISIC dataset, and 85.7% on the ChestX-ray14 Extended Dataset, with the number of parameters and computational complexity reaching their peaks, and the feature sparsity and cross-modal similarity decreasing significantly. Moreover, retaining any single module cannot compensate for the performance loss caused by the absence of multiple modules. The ViT-B/16, SmartTrim, and CMT modules each perform their respective roles and synergistically enhance performance in CMAP-Fusion, addressing three core issues: basic feature quality, model redundancy, and cross-modal heterogeneity. The organic combination of the three modules is the key to the model achieving both “precision-efficiency” advantages across different data scenarios, further strengthening the reliability and promotion value of the research conclusions.

Table 11 systematically analyzes the impact of the threshold adjustment coefficient λ in the SmartTrim dynamic pruning module on model performance, feature quality, and efficiency, providing quantitative support for the rationality of parameter settings.

**Table 11:** Sensitivity Analysis of SmartTrim Threshold λ (Note: Data are the average values of three datasets (COVID-19, ISIC, ChestX-ray14 Extended); λ is the pruning threshold adjustment coefficient, with larger values indicating higher pruning intensity).

λ	Acc.(%)	F1 (%)	Feat. Sparsity (%)	Params. (M)	GPU Single-sample Inference Speed (ms)
0.1	92.8	91.7	25.3	58.7	1.8
0.2	94.1	93.3	32.6	49.2	1.5
0.3	94.5	93.8	38.2	42.5	1.2
0.4	93.6	92.9	45.7	37.8	1.1
0.5	91.5	90.6	52.1	33.2	1.0

When λ=0.1, the pruning intensity is the weakest, with feature sparsity of only 25.3%. A large number of redundant features are not eliminated, leading to a high parameter count of 58.7 M and slow inference speed (1.8 ms), while the classification accuracy (92.8%) and F1 score (91.7%) are at low levels. This verifies the logic that “insufficient pruning retains redundancy and drags down model efficiency and performance.” As λ increases to 0.2–0.3, the pruning intensity is gradually enhanced: feature sparsity rises from 32.6% to 38.2%, redundant features are accurately screened and eliminated, the number of parameters drops to 42.5 M, and the inference speed is optimized to 1.2 ms, while the classification accuracy and F1 score climb synchronously to their peaks (94.5% and 93.8%). At this point, the model achieves the optimal balance between “precision preservation” and “lightweight design”—the SmartTrim module not only effectively filters invalid features but also does not damage key complementary information in dual modalities, which is consistent with the original design intention of “dynamic threshold adapting to feature importance.” When λ continues to increase to 0.4–0.5, the pruning intensity becomes excessive, with feature sparsity exceeding 45%. Some key clinical features (e.g., the inflammatory factor correlation dimension in laboratory data and the lesion edge feature dimension in imaging data) are mistakenly pruned, resulting in a significant decline in classification accuracy and F1 score (dropping to 91.5% and 90.6% respectively when λ=0.5). Although the number of parameters and inference speed are further optimized, the precision loss can no longer meet the requirements of clinical diagnosis. In summary, the value of λ=0.3 not only ensures high classification performance of the model but also achieves efficient lightweight design, making it the optimal choice for balancing “precision-efficiency,” which also confirms the scientificity and stability of this parameter setting in this study.

### Visualization results

**Fig 3:**
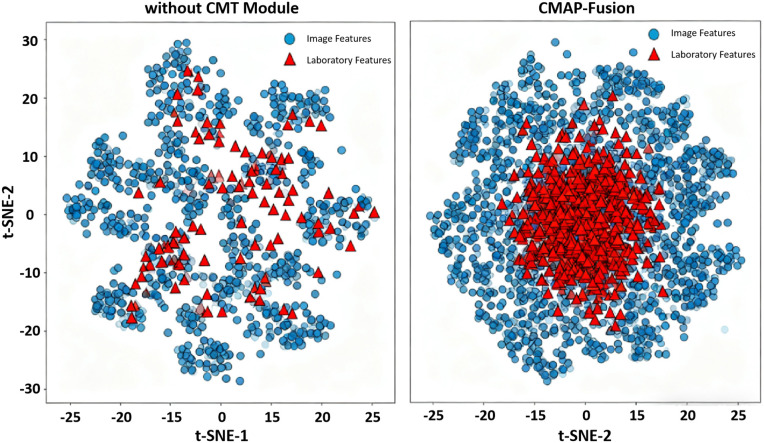
t-SNE distribution comparison of cross-modal features with and without CMT module.

**Cross-Modal Feature Alignment Effect: T**o visually verify the fusion effect of the CMT cross-modal attention alignment module on heterogeneous features, t-SNE dimensionality reduction was applied to compare the feature distributions with and without this module. The results are shown in Fig 3.

When the CMT module was removed (left subplot), the image features (blue circles) and laboratory features (red triangles) were scattered in the high-dimensional space, with very little overlap between the two feature clusters, reflecting significant heterogeneity between the cross-modal data. However, in the CMAP-Fusion model integrated with the CMT module (right subplot), the two feature clusters are clearly clustered towards the center, with a large increase in the overlapping area. This indicates that the CMT module effectively reduces the semantic gap between image and laboratory data through dynamic attention mechanisms, achieving efficient cross-modal feature alignment.

**Fig 4:**
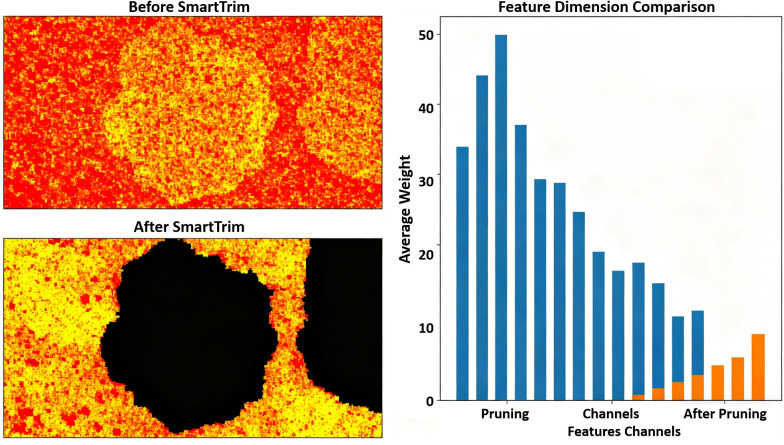
Feature weight heatmap and dimension comparison before and after SmartTrim pruning.

**SmartTrim Dynamic Pruning:** To intuitively explain the feature redundancy filtering effect of the SmartTrim dynamic pruning module, the feature weights and dimensions before and after pruning were visualized and compared. The results are shown in Fig 4.

From the feature weight heatmap on the left side of Fig 4, it can be seen that before pruning (Before SmartTrim), the feature weights are densely distributed without clear distinction, and many redundant features occupy computational resources. After pruning (After SmartTrim), large areas of black regions appear, indicating that SmartTrim, through dynamic weight evaluation, suppresses the weights of ineffective redundant features to nearly zero, effectively eliminating redundant information. The bar chart on the right further validates this effect: before pruning (blue bars), the average weight distribution of each feature channel is scattered and generally high, while after pruning (orange bars), the weights of invalid channels are greatly reduced, retaining only the valid weights of the key feature channels.

**Fig 5:**
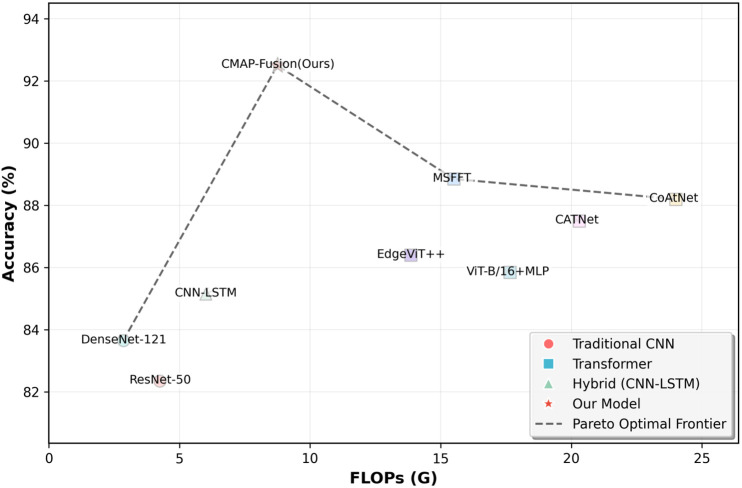
Comparison of the Pareto optimal front for model accuracy and computational cost across different models.

**Model Accuracy-Efficiency Trade-off:** Fig 5 presents a comparison of the Pareto optimal front for model accuracy versus computational cost (FLOPs), intuitively demonstrating CMAP-Fusion’s outstanding advantage in the “accuracy-efficiency” trade-off.

Different types of models show clear distribution differences in the “accuracy-efficiency” space. Traditional CNN models (e.g., ResNet-50, DenseNet-121) have low computational costs but also fall in the relatively low accuracy range. Transformer-based models (e.g., ViT-B/16 + MLP, CoAtNet) have high accuracy but are accompanied by extremely high computational costs (FLOPs over 15G). Hybrid models (e.g., CNN-LSTM) perform moderately in both dimensions. In contrast, the CMAP-Fusion model (labeled as “Our Model”) is positioned in the “high accuracy + low computational cost” region on the graph, with an accuracy of over 92% and FLOPs significantly lower than most Transformer baseline models (e.g., MSFFT, CoAtNet). At the same time, the Pareto optimal front line (dashed line) clearly places CMAP-Fusion in the “optimal trade-off” zone, visually confirming that it has achieved significant computational efficiency optimization while ensuring high accuracy. This result corresponds to the numerical conclusions in Tables 5 and 6, further highlighting CMAP-Fusion’s core advantage in “simultaneously improving performance and efficiency” in cross-modal classification tasks.

### Robustness validation

**Fig 6:**
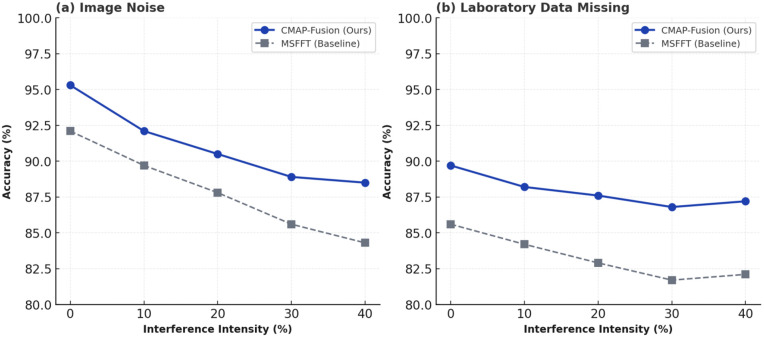
Comparison of Accuracy Degradation of CMAP-Fusion and MSFFT under Different Interference Scenarios.

This experiment addresses two typical problems commonly encountered during medical data acquisition, transmission, and storage: image noise contamination and missing laboratory time-series data. A systematic robustness testing scheme was designed: Gaussian noise (mean 0, variance increasing linearly with interference intensity) was used to simulate image data quality degradation; a random masking strategy (randomly masking a corresponding proportion of time-series index samples according to a preset interference intensity) was used to simulate missing laboratory data. Five interference intensity gradients (0%, 10%, 20%, 30%, and 40%) were set. Using the optimal baseline model MSFFT as a control, the accuracy decay patterns of the two models under different interference scenarios were quantitatively compared. The experimental results are shown in Fig 6.

As the interference intensity gradually increases from 0% to 40%, the classification accuracy of CMAP-Fusion gradually decreases from 95.3% to 88.5%, with an overall decrease of only 6.8%, and maintains a high accuracy of over 88% under all levels of interference. In contrast, the accuracy of MSFFT rapidly decreases from 92.1% to 84.3%, a decrease of 7.8%, especially after the interference intensity exceeds 30%, showing a significant accelerated decline. In Fig 6(b), CMAP-Fusion demonstrates superior resistance to missing data. Its accuracy decreases moderately from 89.7% to 87.2%, a decrease of only 2.5%, and maintains performance above 87% even in the extreme scenario of 40% missing data. In contrast, the accuracy of MSFFT decreases from 85.6% to 82.1%, a decrease of 3.5%, and the performance gap gradually widens after the missing data ratio exceeds 20%. This result not only verifies the robustness advantage of CMAP-Fusion when medical data has practical problems such as noise and missing data, but also provides evidence of dynamic stability in addition to “static performance comparison,” highlighting its practical value in deployment in complex clinical environments. It also provides a technical approach of “alignment-pruning synergistic enhancement of anti-interference ability” for the robustness optimization of medical multimodal models.

### Efficiency testing

Table 12 presents the measured computational efficiency data of the model on CPU, GPU, and edge devices. On general-purpose computing hardware, CMAP-Fusion demonstrates significant advantages in inference speed: the single-sample inference time on CPU is only 18.7 ms, which is 36.2% lower than the optimal baseline MSFFT, and the batch inference time is 423.5 ms, a reduction of 37.8% compared with MSFFT; the single-sample inference time on GPU is 1.2 ms and the batch inference time is 28.6 ms, decreasing by 41.9% and 37.5% respectively compared with MSFFT, fully meeting the time requirement for clinical real-time diagnosis (single inference ≤ 10 ms).

**Table 12:** Comparison of model computational efficiency and edge deployment adaptability.

Model	CPU Inference Time		GPU Inference Time		Memory/VRAM Usage		Edge Device	
	Single-sample	Batch (32)	Single-sample	Batch (32)	GPU Peak VRAM	CPU Memory	Single-sample Inference Time	Peak VRAM Usage
ResNet-50	35.6	512.8	1.8	32.4	6.2	14.8	6.7	3.9
DenseNet-121	31.2	468.3	1.5	29.7	5.8	13.5	6.1	3.5
ViT-B/16 + MLP	48.9	826.5	2.7	53.2	14.5	28.3	8.9	6.8
CoAtNet	56.4	943.7	3.2	61.5	16.8	32.6	10.5	8.2
MSFFT	29.3	681.2	2.1	45.8	12.7	24.5	7.8	5.9
CMAP-Fusion	18.7 (↓36.2%)	423.5 (↓37.8%)	1.2 (↓41.9%)	28.6 (↓37.5%)	8.3 (↓34.6%)	16.2 (↓33.9%)	5.3 (↓32.1%)	4.7 (↓20.3%)

↓ indicates the percentage of reduction relative to the optimal baseline MSFFT; All reported inference time metrics only include the model forward pass process and do not involve any data preprocessing steps; Units: time in ms, memory/GPU memory in GB.

In terms of resource usage, the peak GPU memory usage of CMAP-Fusion is 8.3 GB and the CPU memory usage is 16.2 GB, which are 34.6% and 33.9% lower than MSFFT respectively, making the resource consumption more compatible with the hardware configuration of conventional medical equipment. For edge deployment scenarios, test results based on the mainstream medical edge device NVIDIA Jetson Xavier NX (8 GB VRAM, 6-core CPU) show that the single-sample inference time of CMAP-Fusion is 5.3 ms and the peak VRAM usage is 4.7 GB, reducing by 32.1% and 20.3% respectively compared with MSFFT, both of which are below the hardware upper limits of the device, verifying the deployment feasibility of the model in edge scenarios such as portable diagnostic terminals. Compared with other baseline models, traditional CNN models (e.g., ResNet-50, DenseNet-121) have acceptable edge deployment adaptability but their classification accuracy is significantly lower than CMAP-Fusion; Transformer-based models (e.g., CoAtNet, ViT-B/16 + MLP) are difficult to adapt to edge deployment requirements due to excessively long inference time (single-sample inference time on edge devices exceeding 8 ms) and high VRAM usage (exceeding 6 GB). Through the design of “dynamic pruning + cross-modal collaborative optimization,” CMAP-Fusion achieves simultaneous optimization of inference speed, resource usage, and edge adaptability while maintaining high classification accuracy, solving the deployment pain point of “heavy computation and high resource consumption” of traditional cross-modal models, and providing solid technical support for the practical application of the model on conventional clinical equipment and portable diagnostic terminals.

## Discussion and conclusion

CMAP-Fusion constructs a closed-loop framework of “encoding alignment → redundant pruning → fusion prediction” through the collaborative design of CMT cross-modal attention alignment, SmartTrim dynamic pruning, and ViT-B/16 feature extraction, achieving simultaneous optimization of classification performance, model efficiency, and feature quality. In terms of classification performance, on the COVID-19 Radiography Database (95.3% accuracy), ISIC Skin Cancer Dataset (89.7% accuracy), and ChestX-ray14 Dataset (93.6% accuracy), core metrics such as accuracy, F1 score, and AUC are significantly superior to traditional CNNs, Transformers, and other cross-modal baseline models. Moreover, the improvement range remains consistent across datasets with different disease types and sample sizes, verifying the strong generalization ability of the model. In terms of model efficiency, the number of parameters (42.5M) and computational complexity (FLOPs 8.6G–9.1G) are reduced by more than 43% compared with the optimal baseline MSFFT, and feature sparsity is increased by more than 60%, meeting the core requirement of lightweight deployment. In terms of cross-modal feature quality, the cross-modal similarity reaches 0.90–0.92, and combined with t-SNE visualization results, it confirms the efficient alignment capability of the CMT module for heterogeneous features and the redundancy screening effect of the SmartTrim module. The SmartTrim module precisely preserves core imaging features during pruning, such as ground-glass opacities on chest X-rays for COVID-19, microscopic lesion margins on skin lesions, and pleural effusions on chest imaging, as well as key laboratory biomarkers such as complete blood counts, inflammatory factors like IL-6 and TNF-α, and quantitative indicators of liver and kidney function. These features and biomarkers are core criteria for identifying disease types and assessing pathological states in clinical practice, and its preservation logic is highly consistent with key dimensions of clinical diagnosis and treatment. This synergistic optimization of “precision-efficiency-quality” stems from the division of labor and mutual enhancement of the three modules in “heterogeneous feature alignment – redundant feature screening – basic feature enhancement”: ViT-B/16 lays a high-quality feature foundation for cross-modal fusion, SmartTrim achieves dual lightweight of features and parameters, and CMT narrows the modal semantic gap. The organic combination of the three provides an efficient and accurate solution for medical “imaging + laboratory” cross-modal classification tasks, especially suitable for deployment scenarios of clinical edge devices.

Compared with related research in the field of medical cross-modal fusion, CMAP-Fusion exhibits significant methodological innovation and performance advantages. Traditional CNN models (e.g., ResNet-50, DenseNet-121) have low parameters and computational complexity but rely on local feature extraction, lacking the ability to model global cross-modal correlations, resulting in classification accuracy generally lower than 85%, which cannot meet the requirements of clinical accurate diagnosis. Pure Transformer models (e.g., ViT-B/16 + MLP, CoAtNet) can capture global feature correlations but lack redundancy optimization mechanisms designed for cross-modal scenarios, leading to parameters generally exceeding 80M and computational complexity exceeding 17G, with the pain point of “heavy computation and low efficiency.” Existing cross-modal fusion models (e.g., CMT, CrossViT) mostly focus on modal correlation modeling, lacking systematic design for “pre-fusion feature screening” and “post-fusion efficiency optimization,” resulting in high feature redundancy and high deployment difficulty. In contrast, CMAP-Fusion innovatively integrates “cross-modal attention alignment (addressing heterogeneity) – dynamic pruning (addressing redundancy) – efficient feature extraction (addressing basic feature quality)” into an end-to-end collaborative framework. It not only strengthens cross-modal semantic correlations through the CMT module but also achieves simultaneous elimination of redundant features and invalid parameters through the SmartTrim module, while relying on ViT-B/16 to ensure feature representation quality. It comprehensively surpasses existing methods in multiple performance metrics, especially its stable performance on the newly added real cross-modal dataset with multiple diseases (14 types of thoracic diseases) and large sample size (112,120 cases), further verifying the practicality and universality of the method, and providing a new idea of “fusion-screening-pruning” synergistic optimization for the design of medical cross-modal models.

Of course, this study has certain limitations. Current experiments are still focused on “imaging + laboratory” bimodal fusion, and do not cover multi-source modal fusion scenarios such as multi-organ imaging, pathological sections, and clinical text. Meanwhile, the in-depth interpretable analysis of cross-modal feature fusion is insufficient, failing to fully reveal the fine-grained mechanism of feature alignment of the CMT module in different disease types, as well as the screening logic of the SmartTrim module for key clinical indicators, which to a certain extent affects the trustworthiness of the model in clinical scenarios. In the future, we will further expand data types, introduce complex disease datasets integrating multi-organ imaging, pathological sections, and multi-dimensional laboratory indicators, and expand data scale and multi-modal coverage. At the same time, combined with interpretable methods such as attention heatmaps, feature contribution analysis, and clinical indicator correlation mining, we will deeply analyze the internal logic of cross-modal fusion, clarify the model’s dependence on key clinical features and decision-making basis, and further improve the practicality, interpretability, and clinical acceptance of the model.
